# Why Are Inequalities in Disability-Free Life Expectancy by Socioeconomic Position Widening?

**DOI:** 10.1093/geroni/igaa057.2195

**Published:** 2020-12-16

**Authors:** Carol Jagger

## Abstract

Life expectancy has increased over previous decades, but several countries are seeing widening inequalities in disability-free life expectancy (DFLE) by socioeconomic position (SEP). In this symposium we address three unanswered questions.1. Do DFLE trends differ for SEP groups, and which of the underlying transitions (incidence, recovery, death when disability-free, death when already disabled) explains the differences?2. Do DFLE trends by SEP depend on when in the life-course SEP is measured (early life - education, mid-life - occupational status or late-life - material disadvantage)?3. How much does multi-morbidity contribute to differing trends in DFLE by SEP, since multi-morbidity is more prevalent in low SEP groups? To answer these questions, we use unique longitudinal studies of older people across different generations in two countries: the UK (Cognitive Function and Ageing Studies – CFAS I and II) and Australia (Household, Income and Labour Dynamics in Australia – HILDA). The first presentation sets the scene with findings from a systematic review of worldwide trends in life and healthy life expectancy by SEP. Presentations two and three examine the first question using DFLE at age65 by SEP defined by late-life disadvantage in CFAS (1991-2011), followed by HILDA (2001-2017). The fourth presentation investigates the effect of different life-course measures of SEP using HILDA. The final presentation from CFAS examines the third question. This symposium increases our understanding of how and why inequalities in DFLE by SEP are changing with the goal of achieving healthy ageing for all.

